# Simple, biologically-constrained CA1 pyramidal cell models using an intact, whole hippocampus context

**DOI:** 10.12688/f1000research.3894.1

**Published:** 2014-05-09

**Authors:** Katie A. Ferguson, Carey Y. L. Huh, Benedicte Amilhon, Sylvain Williams, Frances K. Skinner

**Affiliations:** 1 Toronto Western Research Institute, University Health Network, Toronto, Ontario, M5T 2S8, Canada; 2 Department of Physiology, University of Toronto, Toronto, Ontario, M5S 1A1, Canada; 3 Department of Psychiatry, Douglas Mental Health University Institute, McGill University, Montreal, Quebec, H4G 1X6, Canada; 4 Department of Medicine (Neurology), Physiology, University of Toronto, Toronto, Ontario, M5S 1A1, Canada

## Abstract

The hippocampus is a heavily studied brain structure due to its involvement in learning and memory. Detailed models of excitatory, pyramidal cells in hippocampus have been developed using a range of experimental data. These models have been used to help us understand, for example, the effects of synaptic integration and voltage gated channel densities and distributions on cellular responses. However, these cellular outputs need to be considered from the perspective of the networks in which they are embedded. Using modeling approaches, if cellular representations are too detailed, it quickly becomes computationally unwieldy to explore large network simulations. Thus, simple models are preferable, but at the same time they need to have a clear, experimental basis so as to allow physiologically based understandings to emerge. In this article, we describe the development of simple models of CA1 pyramidal cells, as derived in a well-defined experimental context of an intact, whole hippocampus preparation expressing population oscillations. These models are based on the intrinsic properties and frequency-current profiles of CA1 pyramidal cells, and can be used to build, fully examine, and analyze large networks.

## Introduction

Networks of excitatory and inhibitory neurons are essential components constituting the functional structures of our brains. Dysfunction is thought to occur when inappropriate excitation-inhibition balances occur ^1^. From a modeling perspective, these balances are determined by the choice of parameters in the equations representing neurons and networks. Mathematical models of neurons and networks are developed so that they can be used to determine the mechanisms underlying brain functions. However, it is difficult to assess whether the mechanisms determined from mathematical models actually occur biologically ^2, 3^. Furthermore, it is well known that cellular models used in building network models affect and can dictate the network output ^4, 5^. To address this recognized difficulty we are developing models that are based on well-defined experimental contexts in which both the cellular and the network aspects of the model can be considered simultaneously ^6, 7^. Using such models, we aim to help determine, predict and test biologically based mechanisms.

Here we use a well-defined experimental context of an *in vitro* intact, whole hippocampus preparation in which spontaneous population theta and theta-gamma rhythms are expressed ^8, 9^. Access to many cellular details is possible *in vitro*, and with a physiologically relevant output of theta and theta-gamma rhythms, a reasonable and functional scenario is also present. We have previously developed models of CA1 fast-spiking inhibitory cells in the same experimental context, and used them to show that model inhibitory networks of these fast-spiking cells exhibit sharp transitions between random and coherent firings at high frequencies (>90 Hz) when connectivity constraints were imposed ^6^. In this article, we present the result of CA1 pyramidal cell models developed in the same context of this *in vitro* whole hippocampus preparation. Similar to our previous cellular model developments ^6^, the models presented here are biologically-based at the cellular level, but do not have a biophysical representation in terms of conductance-based model representations. These CA1 models use a simple two variable mathematical formulation based on Izhikevich ^10^ and they include rebound firing and adaptation characteristics. Using these simple, yet biologically-based models, we can build and examine several large network models that are aligned with the biology. Subsequent analyses of these large network models could determine the mechanisms by which particular cellular characteristics critically contribute to the population activities observed in our experimental context.

## Methods

### Experimental data

***Animals.*** Three mice (two female, post-natal day 20–29) were used. We used transgenic mice that expressed a fluorescent protein, tdTomato, under the control of the PV promoter. PV-Cre homozygote mice (strain name: B6;129P2-Pvalb ^tm1(cre)Arbr^/J, stock number: 008069, Jackson Laboratory) were mated with a reporter line, Ai9 homozygote mice (strain name: B6;129S6-Gt(ROSA)26Sor ^tm9(CAG-tdTomato)Hze^/J, stock number: 007905, Jackson Laboratory) to generate PV-tdTomato mice. The mouse lines are on genetic backgrounds that are mixtures of C57BL/6 and a type of 129 mice; PV-Cre mice are C57BL/6;129P2 and Ai9 mice are C57BL/6;129S6. Mice were bred in-house at the Douglas animal facility and kept in standard laboratory cages with standard bedding and environmental enrichment. They were housed in a temperature-controlled room with a 12:12 hours dark/light cycle with food and water provided ad libitum. All animals were treated according to protocols and guidelines approved by McGill University and the Canadian Council of Animal Care (CCAC). Ethical approval was obtained to conduct this study (approval number: 2010-5827). The authors note that the use of scissors to decapitate mice at that age without administering anesthesia (which could have altered synaptic transmission) was approved by the CCAC.

***Intact hippocampal preparation.*** The acute preparation containing the whole hippocampus was obtained from PV-tdTomato mice according to a previously described protocol ^8^. Briefly, after decapitation using scissors, the brain was quickly removed from the skull and placed in ice-cold high-sucrose solution, containing (in mM) 252 sucrose, 24 NaHCO _3_, 10 glucose, 3 KCl, 2 MgCl _2_, 1.25 NaH _2_PO _4_ and 1 CaCl _2_ (pH 7.3, oxygenated with 95% O _2_-5% CO _2_). From a hemisected brain, the septum and hippocampus along with the interconnecting fibers were carefully and rapidly dissected out using a microspatula. The preparation was trimmed in ice-cold high-sucrose solution (same contents as the high-sucrose solution listed above) using fine scissors to remove any remaining cortical tissue and the septum. Then, the surface of the preparation was cut at a ~45° angle to expose the pyramidal layer of CA1. The cut enabled visually guided patch-clamp recordings of pyramidal cells which yielded a higher success rate for whole-cell recordings compared to the blind-patch technique used previously ^8^. The visual approach allowed identification of CA1 pyramidal cells by their soma location, morphology and the lack of PV-tdTomato fluorescence in the soma. The hippocampal preparation was then left to equilibrate in oxygenated room-temperature high-sucrose solution for 30 min - 1 h before recording. The preparation from only one hemisphere was used for recording from each mouse, and the preparation from either the left or the right hemisphere was chosen randomly for each experiment. Three mice were used in total; we used three intact hippocampal preparations from these mice (one from each mouse) and one pyramidal cell was recorded from each preparation, except for one preparation from which two cells were recorded.

All electrophysiological recordings were performed at 30 ± 2ºC, using artificial cerebrospinal fluid (aCSF) containing (in mM) 126 NaCl, 24 NaHCO _3_, 10 glucose, 4.5 KCl, 2 MgSO _4_, 1.25 NaH _2_PO _4_, 0.4 ascorbic acid and 2 CaCl _2_ (pH 7.3, oxygenated with 95% O _2_-5% CO _2_). The hippocampal preparation was placed in a custom-made submerged recording chamber lined with a nylon mesh, and firmly stabilized by carefully placing several lead weights at both septal and temporal poles of the hippocampal preparation. We placed the hippocampal preparation in the recording chamber, such that the CA1 was the most superficial and accessible sub-region for visualization and whole-cell recordings. Recordings were restricted to neurons located within the middle portion of the hippocampus (intermediate between septal and temporal poles of the preparation). The preparation’s stability in the recording chamber was extremely important as aCSF was perfused at the rate of 20–25 ml/min during recordings. Since the tissue is several millimeters thick, such a fast perfusion rate is necessary to ensure sufficient oxygenation. This fast perfusion rate is also required to generate intrinsic theta oscillations from intact hippocampal preparations ^8^. In order to achieve temperature stability, aCSF was pre-heated using an electric skillet and further regulated via an automatic temperature controller (Warner Instruments, Hamden, CT). The electrophysiology setup was equipped with an upright BX51W1 Olympus microscope, a 20× water-immersion objective, Nomarsky optics, an infrared camera (Cohu, San Diego, CA), a variable-wavelength fluorescence system (PTI, Monmouth Junction, NJ) and a monochrome digital camera for fluorescence imaging (DAGE-MTI, Michigan City, IN). Patch pipettes were pulled from borosilicate glass capillaries (2.5–4 MΩ) and filled with the intra-pipette solution containing (in mM) 144 K-gluconate, 10 HEPES, 3 MgCl _2_, 2 Na _2_ATP, 0.3 GTP, 0.2 EGTA, adjusted to pH 7.2 with KOH. The patch electrode was controlled using a motorized micromanipulator (Sutter Instruments, Novato, CA). An Axopatch-1C amplifier (Axon Instruments, Foster City, CA) and pClamp9 software (Molecular Devices, Sunnyvale, CA) were used for recording. The junction potential was estimated at -15.2 mV and was corrected for. All drugs were obtained from Sigma-Aldrich (St. Louis, MO), unless otherwise noted.

***Intrinsic property characterization.*** Intrinsic properties were characterized in current-clamp mode following published protocols with minor modifications ^11^. A mixture of synaptic blockers was used to inhibit synaptic activity: 5 μM 6,7-dinitroquinoxaline-2,3-dione disodium salt (DNQX), 5 μM bicuculline and 25 μM DL-2-amino-5-phosphonopentanoic acid sodium salt (DL-AP5) (Abcam, Toronto, Canada). Data analysis was done using custom Matlab software (MathWorks, Natick, MA). Once the whole-cell configuration was achieved, the cell’s resting membrane potential was noted and its spontaneous firing, if any, was recorded for 30 s. Access resistance and resting membrane potential were checked at regular intervals (every ~5 min) throughout the recording of the cell.

The frequency-current (f-I) profiles of the pyramidal cells are important to characterize, as we aim for our single cell model to respond to a variety of synaptic input strengths with frequencies similar to that observed experimentally. These f-I curves were determined by applying depolarizing current steps of 1 s duration to cells held in current clamp. Amplitudes were increased incrementally with step sizes of 25 pA for one of four cells, and 10 pA for three of four cells. The initial firing frequency was determined based on the inverse of the first inter-spike interval, and the final frequency was based on the inverse of the last inter-spike interval in the 1-second depolarizing step. For each cell, the approximate linear slope of the f-I curve above 5 Hz was determined using a least squares method. These values were chosen since above 5 Hz the slope was well-approximated by linearization. In addition, the minimum amount of current required to initiate a spike, the rheobase current ( *I _rheo_* in pA), was determined. The action potential threshold was set to be the first voltage point such that the slope of the membrane potential exceeded 20 mV/ms ^12^. The spike width was determined at the threshold value. In addition, the spike height from threshold and the minimum membrane potential reached following the spike were measured. Recordings were kept for analysis only if the neuron remained stationary; spikes overshot 0 mV (-15 mV junction potential corrected) and access resistance < 30 MΩ.

### Mathematical model

We built a pyramidal cell model based on Izhikevich’s ^10^ simple spiking model structure. We chose this model as it captures the cell’s ability to produce rebound spiking, the approximate spike shape, and the frequency-current profile of the cell, including spike-adaptation. Thus, this model is relatively simple, but allows one to capture important biophysical properties of pyramidal cells.

The model has a fast variable representing the membrane potential, *V* ( *mV*), and a slow “recovery” current given by the variable *u* ( *pA*). In order to capture the spike width at threshold, we slightly modified the Izhikevich model by using a different “ *k*” parameter above and below the spike threshold ( *k _high_* and *k _low_* respectively). The model is given by:

*C _m_* $\dot{V}$ = *k*( *V–v _r_*) ( *V–v _t_*) – *u* + *I _applied_*

$\dot{u}$ = *a*[ *b*( *V – v _r_*) – *u*]                                           (1)

*if* *V* ≥ *v _peak_,* *then* *V* ← *c*, *u* ← *u* + *d*

*Where* *k = k _low_* *if* *V* ≤ *v _t_* ; *k = k _high_* *if* *V* > *v _t_*

The parameters are as follows:

*C _m_* ( *pF*) is the membrane capacitance.

*v _r_* ( *mV*) is the resting membrane potential.

*v _t_* ( *mV*) is the instantaneous threshold potential.

*v _peak_* ( *mV*) is the spike cut-off value.

*I _applied_* ( *pA*) is the applied current, and represents the applied input into the cell.

*a* ( *ms* ^-1^) is the recovery time constant of the adaptation current.

*b* ( *nS*) describes the sensitivity of the adaptation current to subthreshold fluctuations. Greater values couple *V* and *u* more strongly resulting in possible subthreshold oscillations and low-threshold spiking dynamics. Further, the sign of *b* determines whether the effect of *u* is amplifying ( *b* < 0) or resonant ( *b* > 0).

*c* ( *mV*) is the voltage reset value.

*d* ( *pA*) is the total amount of outward minus inward currents activated during the spike and affecting the after-spike behaviour.

*k* ( *nS/mV*) represents a scaling factor. *k _high_* is used to adjust the spike width after the threshold.

As with the experimental frequency-current (f-I) curve frequencies, we exhibited the “initial” and “final” f-I curves for each model, where the initial curves were based on the inverse of the first inter-spike interval (ISI) due to a one second current step, and the final curves were based on the inverse of the last ISI. A linear fit based on the least squares method (on frequencies over 10 Hz) was done for each curve (using 5 Hz gave essentially the same results). We then chose parameters in which our models exhibited similar initial and final f-I curves to those of the experimental pyramidal cells. To do so, we first set the resting membrane potential at the rheobase, the spike threshold, the minimum potential reached by the spike after-hyperpolarization, the spike peak, and the spike width at threshold (providing us with values for *v _r_*, *v _t_*, *c*, *v _peak_*, and *k _high_* respectively) based on our experimentally determined values (see Results). The threshold is defined in “Intrinsic Property Characterization”. Since our pyramidal cells exhibited resonant properties, we considered *b* values such that *b* > 0. We initially held *b* and *k _low_* constant (at 0.2 nS and 0.05 nS/mV), and varied *a* between 0 and 1 with an initial step size of 0.01, and *d* between 0 and 20 with a step size of 1. Choosing our *a* and *d* parameters that returned the best fits to our initial and final slopes, we then varied *b* between 0.1 and 10, and *k _low_* between 0 and 20 (both with a step size of 0.1). Noting that we required more adaptation, we then returned to vary *a* between 0 and 0.1 with an initial step size of 0.0001, and *d* again between 0 and 20 with a step size of 1.

## Results

Using the described whole hippocampal preparation and recordings from four CA1 pyramidal cells (from three mice), we developed our simple biologically-based cellular models. Our goal is to obtain representative CA1 pyramidal cell models that capture the essence of the experimental data in the described experimental context. By biologically-based, we mean that passive properties and spike characteristics are captured. Specifically, resting potential, spike threshold, spike width, spike peak and after-hyperpolarization potentials were incorporated into our models. In addition, the adaptation characteristics determined from the frequency-current (f-I) curves (see Methods) are captured. Although not directly characterized with experimental recordings here, our models express rebound firing. Rebound firing (from inhibition or hyperpolarization) has been shown in several experimental studies ^13, 14^ and is an important consideration in population theta activities.

**Figure 1.**
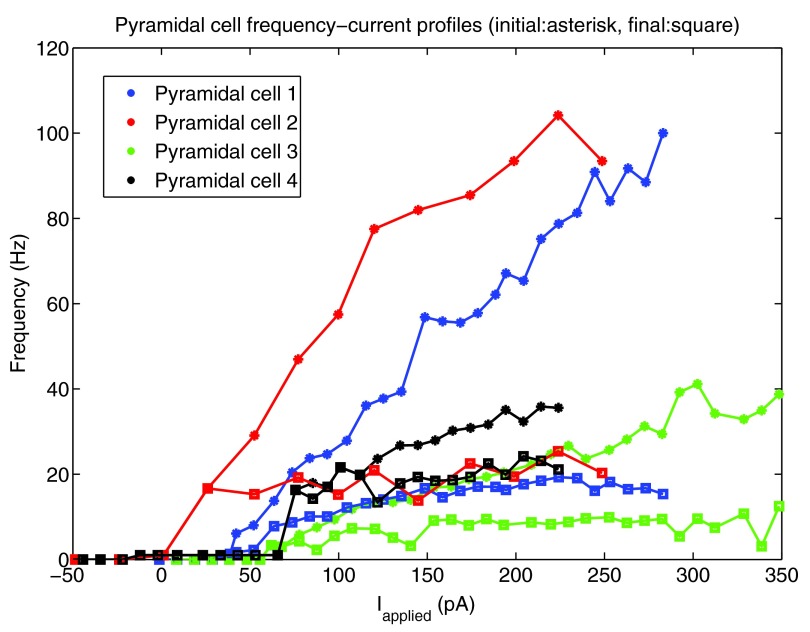
The initial and final frequency current profiles for the four pyramidal cell recordings (from three mice) in the CA1 region of the intact hippocampal preparation *in vitro*. 10 pA depolarizing steps were taken for all cells except Pyramidal cell 2 (25 pA steps). The initial (final) frequencies are shown by asterisks (squares), and the lines interpolate between the data points. Pyramidal cells 1 and 2 (shown in blue and red) have higher initial frequencies, and exhibit more adaptation than Pyramidal cells 3 and 4 (shown in light green and black).

Analysis of the experimental data yielded the following: at rheobase, the pyramidal cell membrane potential rested at -61.8 ± 2.9 mV (mean ± SEM), the spike threshold was -57.0 ± 2.2 mV, the after hyperpolarizing potential reached a minimum of -65.8 ± 3.83 mV, and the spike reached a peak at 22.6 ± 19.9 mV (n=4 cells). The spike width at threshold, was 3.6 ± 0.48 ms. Thus, we set *v _r_* = -61.8 mV, *v _t_* = -57.0 mV, *c* = -65.8 mV, *v _peak_* = 22.6 mV, and *k _high_* = 3.3 nS/mV in our models (see Methods). The remaining model parameters were chosen such that the rheobase and f-I curves of the cell model were similar to those of the cell recordings. To do so, we first considered the f-I curves of the cell recordings ( Figure 1). To demonstrate the amount of adaptation that the cell exhibited, we created two f-I curves for each cell: one based on the first inter-spike interval (ISI) of the cell’s spiking during a one second current step (denoted initial curve, data points shown as asterisks in Figure 1), and one based on the last (final curve, data points shown as squares in Figure 1). If the cell only had one spike in the 1s trace, a frequency of 1 Hz was given. We note that two of our recordings (Pyramidal cell 1 and Pyramidal cell 2, shown in red and blue in Figure 1) exhibited stronger adaptation than the other two (Pyramidal cell 3 and 4, shown in green and black in Figure 1). Thus, we created pyramidal cell models based on the strongly adapting cells and the weakly adapting cells separately. While adaptation clearly exists (e.g., here, ^15, 16^), biological variability, precise protocols and contexts need to be considered. As such, we did not aim to *exactly* capture adaptation characteristics, but rather to capture strongly and weakly adapting cells as represented by the data here. Also, since our model has a simple, mathematical structure, it is limited in the extent of biologically-based characteristics that it can encompass.

### Strongly adapting model

**Figure 2.**
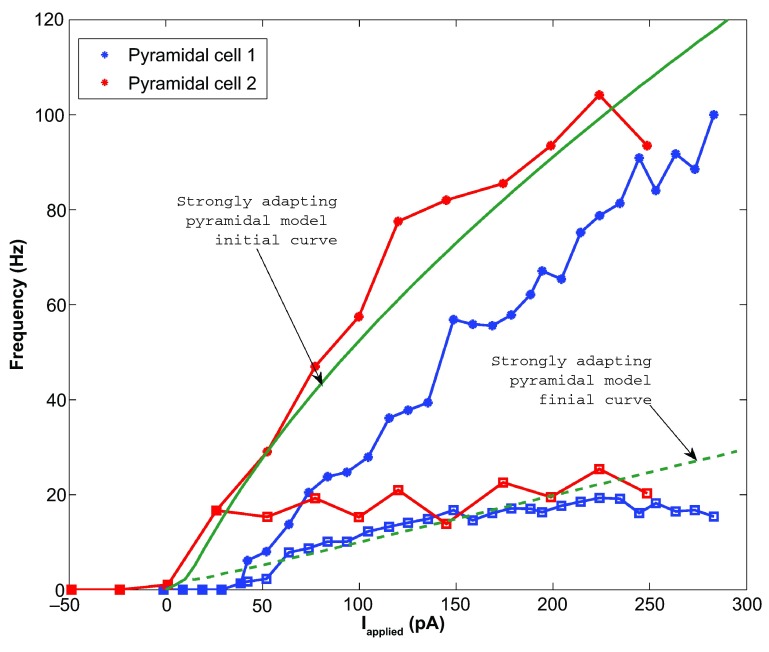
The strongly adapting pyramidal cell f-I curves (Pyramidal cells 1 and 2 shown in blue and red respectively) are shown against the strongly adapting pyramidal cell model (dark green). The initial data points are shown as asterisks and the final points shown as squares. The initial model curve is shown as a solid line (initial slope: 0.432), and the final curve shown as a dashed line (final slope: 0.099). The model rheobase (~0 pA) and slope approximate those determined experimentally.

It is important that our model f-I curve captures two important properties of the experimental data: the rheobase current (i.e. the minimum amount of current required to initiate a spike), and the approximate slope of the curve. If these properties are captured, then the model will spike with similar frequencies as the physiological cell given the same amount of synaptic input. We found that for the two strongly adapting pyramidal cells (1 and 2), the slope of the linear least squares approximation of the f-I curves above 5 Hz were 0.376 and 0.385 for the initial curves, and 0.030 and 0.040 for the final curves. Again, the initial frequencies are based on the inverse of the first inter-spike interval due to a one second current step, and the final frequencies are based on the last inter-spike interval. A series of depolarizing (25 and 10 pA) steps were used to determine the rheobase currents, which were 1.2 pA and 38.7 pA for the two strongly adapting cells (f-I curves, red and blue curves in Figure 1 or data in Figure 2).

**Figure 3.**
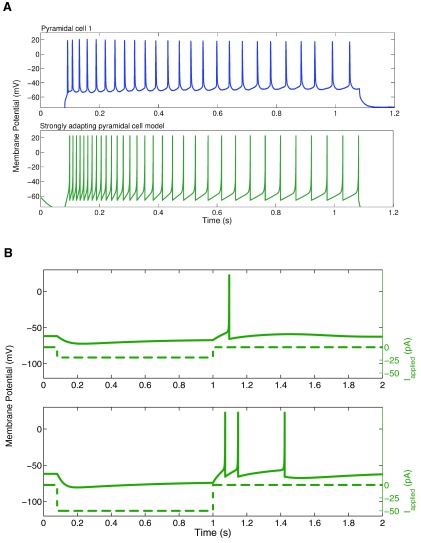
**A**: An example intracellular recording of Pyramidal cell 1 during current clamp with applied current of 188 pA (top, blue) is compared with the firing of our strongly adapting pyramidal cell model, also with an applied current of 188 pA (bottom, dark green). The firing rates and amount of adaptation of the model are similar to those of the experiment. **B**: Two examples of rebound firing in our strongly adapting model. In each case, a one-second step of hyperpolarizing input is applied (shown as dashed lines; top: 20 pA step, bottom: 50 pA step). In each case, the strongly adapting model produces rebound spiking (solid line), where more spiking occurs for larger amounts of hyperpolarizing input.

We kept our previously determined parameters constant (i.e. *v _r_* = -61.8 mV, *v _t_* = -57.0 mV, *c* = -65.8 mV, *v _peak_* = 22.6 mV, and *k _high_* = 3.3 nS/mV), and set our membrane capacitance to *C _m_* = 115 *pA*. We then varied the parameters *a, b, d,* and *k _low_* to produce multiple models. We determined the rheobase current and the slope of the initial and final f-I curve over 10 Hz (using a least squares approach) for each model in order to settle upon a final model in which our initial and final f-I slopes and rheobase approximated that which we determined biologically. We determined that *a* = 0.0012 *ms* ^-1^ *, b* = 3 *nS, k _low_* = 0.1 *nS/mV*, and *d* = 10 *pA*. This gave us a model f-I initial slope of 0.432, a final slope of 0.099, and a rheobase of ~0 pA (see Figure 2). As shown in Figure 3, the model shows strongly adapting firing ( Figure 3A) and rebound firing when hyperpolarized ( Figure 3B).

### Weakly adapting models

Following a similar methodology, we created two separate models for the weakly adapting pyramidal cells: the first better captures the cell’s weak adaptation, especially for larger currents, but has a steep final slope, whereas the second captures the more gradual slope of the final f-I curve, but doesn’t exhibit the cell’s weak level of adaptation.

**Figure 4.**
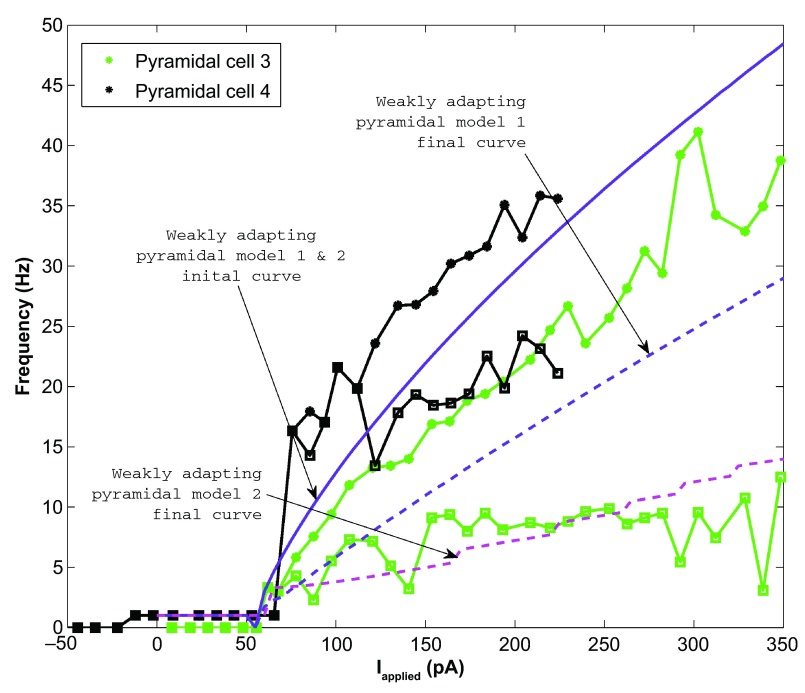
The weakly adapting pyramidal cell f-I curves (Pyramidal cells 3 and 4 shown in green and black respectively) are shown against the weakly adapting pyramidal cell models (model 1: purple; model 2: purple solid line and magenta dashed line). The initial data points are shown as asterisks and the final points shown as squares. The initial model curve is shown as a solid line (initial slope: 0.136), and the final curves are shown as dashed lines. Model 1 exhibits less adaptation (final slope for model 1: 0.089; final slope for model 2: 0.048), but higher final frequencies than model 2 (compare purple and magenta dashed lines). The model rheobase (5 pA) and slopes approximate those determined experimentally.

**Figure 5.**
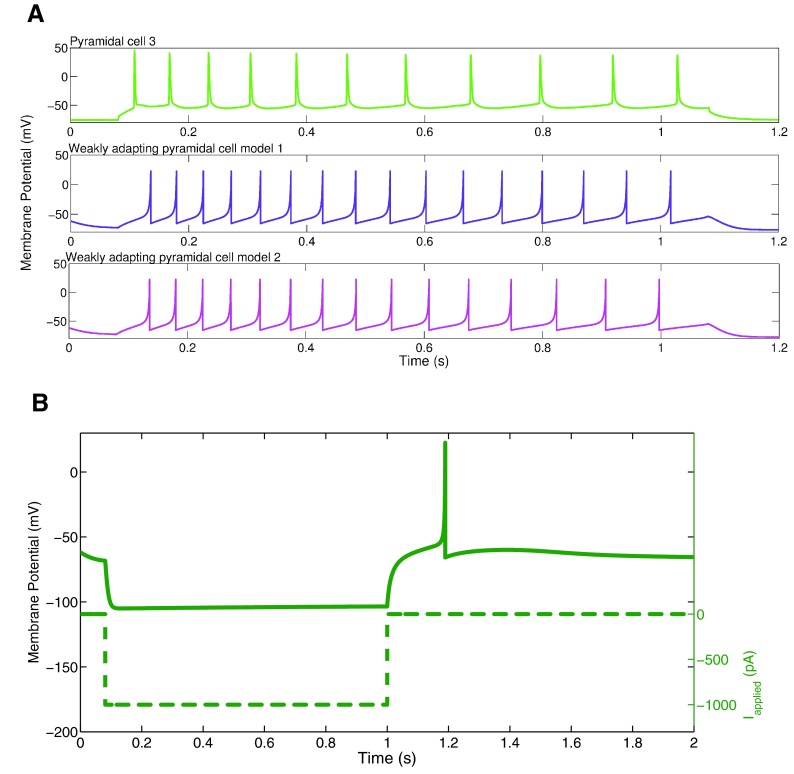
**A**: An example intracellular recording of Pyramidal cell 3 during current clamp with applied current of 154 pA (top, light green) is compared with the firing of our weakly adapting pyramidal cell models, also with an applied current of 154 pA (model 1: middle, purple; model 2: bottom, magenta). The firing rates and amount of adaptation of the model are similar to those of the experiment. **B**: An example of rebound firing in our weakly adapting model 1. A one-second step of 1000 pA hyperpolarizing input is applied (dashed line). The weakly adapting model produces rebound spiking (solid line), but requires a reasonably large amount of applied input. The weakly adapting model 2 does not produce rebound spiking following steps of hyperpolarizing input in the physiological range.

We found that for the two weakly adapting pyramidal cells, the slope of the linear least squares approximation of the f-I curves were 0.119 and 0.138 for the initial curves, and 0.013 and 0.044 for the final curves (values relating to experimental, data green and black in Figure 1 and Figure 4). A series of depolarizing (10 pA) steps were used to precisely determine the rheobase currents, which were 62.0 pA and -12.1 pA for the two weakly adapting cells. We kept our previously determined parameters constant (i.e. *v _r_* = -61.8 mV, *v _t_* = -57.0 mV, *c* = -65.8 mV, *v _peak_* = 22.6 mV, and *k _high_* = 3.3 nS/mV), and set our membrane capacitance to *C _m_* = 300 *pA*, which allowed us to obtain the gradual f-I slope. We then varied the parameters *a, b, d,* and *k _low_* to produce multiple models, and again determined the rheobase current and the slope of the initial and final f-I curve over 10 Hz (using a least squares approach) for each model. In addition, to obtain an appropriate rheobase current, we included a shift in the applied current ( *I _applied_* → *I _applied_* + *I _Shift_*). For our first model, we determined that *a* = 0.001 *ms* ^-1^ *, b* = 3 *nS, k _low_* = 0.5 *nS/mV*, *d* = 5 *pA*, and *I _shift_* = –45 *pA*. This gave us a model f-I initial slope of 0.136, a final slope of 0.089, and a rheobase of 5 pA (see purple solid and dashed lines in Figure 4). The second weakly adapting model is identical to the first, except that we explored smaller *a* parameter values in order to capture the gradual slope of the final f-I curve. For this model, *a* = 0.00008 *ms* ^-1^, which gave an initial f-I slope of 0.136, a final slope of 0.048, and a rheobase of 5 pA (see purple solid and magenta dashed lines in Figure 4). An example of the weak adaptation in this case is shown in Figure 5A, and rebound firing for model 1 is shown in Figure 5B.

## Discussion and conclusion

We have developed simple, biologically-based cellular models of pyramidal cells from CA1 hippocampus. These models capture the frequency-current profiles of both strongly and weakly adapting cells. Importantly, we have used pyramidal cell recordings from a well-defined experimental context as a basis for our model development. We note that even though other simple pyramidal cell models exist with a conductance-based biophysical representation ^17^, there is not a clear link with adaptation or rebound firing characteristics as exists in pyramidal cells. We note that while modifications have been done to such simple models (e.g., Stark *et al.* ^18^ used the Olfusen *et al.* model ^19^ which incorporated sodium currents, potassium current, voltage-dependent M currents, and a leak current, and added an h-current based on ^20^ to incorporate rebound capabilities), the resulting cellular model outputs constraints with respect to experimental data are unclear, and as such, we consider them not to be biologically-based *per se*.

Our models do not include the multiple voltage-gated channels known to be present in pyramidal cells, and are not spatially extended by being multi-compartment in nature. Multi-compartment, biophysical models of CA1 pyramidal cells exist ^21^ as well as single-compartment biophysical models ^22^ with various voltage-gated channels, but the goals in developing those models are different. Here, we are interested in capturing cellular characteristics in a well-defined experimental context (for subsequent large network explorations that take advantage of the experimental context), whereas these other studies have considered, for example, conductance balances to understand how they contribute to cellular output. We further note that the richness of detail in CA1 pyramidal cells is expanding (e.g., see ^16^ in which countermodulation by metabotropic receptors in bursting or regular spiking pyramidal cells was shown). In essence, it is always the case that the mathematical models are a limited representation of the biology.

In previous work, we used adaptation characteristics from the literature to develop simple models of CA3 pyramidal cell models, and showed that population bursting could occur in excitatory networks if the adaptation characteristics were in line with the experimental data ^23^. In this work we had a full set of experimental recordings and so could capture appropriate cellular characteristics more directly. Although there should not be large differences in some cellular characteristics (e.g., spike widths etc.), there could be differences in characteristics such as rheobase and adaptation amounts due to varying experimental contexts (e.g., solutions, recording setup details and so on – see ^13– 16^). Coupled with biological variability, it would be additionally challenging to be clear about model limitations in subsequent model usage. Here, with our simple mathematical model representation and knowledge of the biological variability in hand, one is easily aware of any changes in the parameters of the model that would result in large deviations from the experimental data.

We note that robust fitting strategies of experimental data to simple, mathematical representations of neurons are being developed ^24^. However, there are some differences in the modeling goals. In the paper by Hertäg *et al.* ^24^, the goal was to use the developed models based on *in vitro* recordings to predict spiking in an *in vivo* context. Here, we have developed our cellular models in the experimental context for which we build the network models to determine physiologically-based mechanisms. One can consider using our models in other contexts, keeping in mind the limitations associated with the models as developed. In essence, when incorporating various cellular characteristics, one should express the choices, rationale and reasoning behind the model development, which naturally stem from the modeling goals.

In conclusion, with our simple, developed models as presented here, we can proceed to considering very large networks that include these models in this experimental context. Furthermore, our simple model representations will also allow us to take advantage of developed theoretical analyses ^25, 26^.

## Data availability

ZENODO: Data set of CA1 pyramidal cell models using an intact whole hippocampus preparation, DOI: 10.5281/zenodo.8747 ^27^
