# Supplementary material for: Simple, biologically-constrained CA1 pyramidal cell models using an intact, whole hippocampus context
Source: F1000Res. 2014 May 9;3:104. [Version 1] doi: 10.12688/f1000research.3894.1 (PMC4215760; doi:10.12688/f1000research.3894.1)
Supplement: Peer review history

# Peer review: Referee response for version 1

**DOI:** 10.5256/f1000research.4170.r4729

**Published:** 2014-06-24

**Authors:** Alla Borisyuk

**Affiliations:** 1 Department of Mathematics, University of Utah, Salt Lake City, UT, USA

The article presents minimal models for CA1 pyramidal cells, based on fitting the frequency-current curves that had been experimentally recorded in whole hippocampus preparation.

There are two types of models, differing in some parameter values, to account for “strongly” and “weakly” adapting cell varieties. The goal of the present work is to present the models and their basic fit to single cell data, in preparation for future large network studies.

Comments:

I think this work would be much stronger if testable predictions were generated from the models. For example, what would be the responses to time varying inputs and how the responses would differ between  the strongly and weakly adapting cells?

It is not clear to me that obtaining one model for each of variety of cells is sufficient. For example, two strongly adapting cells have very different rheobase current values. If you fit the model to each cell individually, I expect that you will end up with two very different parameter sets. What would be the consequences for responses to time varying inputs, for example? Considerations like these will be very important when you decide how much variability and in which parameters to include in the  network model. This is also related to the fact that a single model was not able to capture all desired properties of the weakly adapting model. I would like to see more discussion on the consequences of the spectrum of models for future network work.

I have read this submission. I believe that I have an appropriate level of expertise to confirm that it is of an acceptable scientific standard.

# Response

**Published:** 2014-06-24

**Authors:** Frances Skinner

**Affiliations:** University Health Network, Canada

We would like to thank the reviewer for their helpful comments, which we will be taking into consideration.

We would like to point out that we focused on the single cell model and did not try to also include network simulations in this article as it was originally submitted as a 'Short Research Article' type.

However, *F1000Research* recently replaced the 'Short Research Article' category with a shorter 'Research Notes' article type. As this article did not fit the criteria of a 'Research Note', it appears as a 'Research Article' instead.

# Peer review: Referee response for version 1

**DOI:** 10.5256/f1000research.4170.r4728

**Published:** 2014-06-05

**Authors:** Farzan Nadim, Haroon Anwar

**Affiliations:** 1 Department of Biological Sciences, New Jersey Institute of Technology, Newark, NJ, USA

A simple mathematical yet biological model of hippocampal CA1 pyramidal cell has been developed to capture the cellular characteristics (f-I curve, rheobase current, firing rate adaptation and rebound spiking) of those neurons in an intact preparation of hippocampus. 

Although this study does not examine any biological question or hypothesis directly, the simplified models of CA1 pyramidal cells, in conjunction with a similar model of the fast spiking interneurons previously developed by this lab, can be used in future modeling studies that explore the network properties in the hippocampus.  

Comments:

1. First paragraph of the Introduction:
*"To address this recognized difficulty we are developing models that are based on well-defined experimental contexts in which both the cellular and the network aspects of the models can be considered simultaneously." *
**Please specify more clearly about the network aspects, you are referring to.**
2. First paragraph of Introduction:
*"Using such models, we aim to help determine, predict and test biologically based mechanisms."*
**This is a bit misleading. In this work, the model only replicates biological behavior of neurons. There is no clear prediction of any biological phenomenon.**
3. End of the second paragraph of Introduction:
*"we can build and examine several large network models that are aligned with the biology. Subsequent analyses of these large network models could determine the mechanisms by which particular cellular characteristics critically contribute to the population activities observed in our experimental context." *
**It would be more exciting to construct network models and make biological predictions. Construction of simple cellular models to capture biological characteristics of individual neurons is not novel. In fact, the authors have used similar modeling approach to develop models in references 6 and 23.**
4. Second paragraph of Results - Stronger adaptation vs weaker adaptation - **Has it been shown before? If yes, please include some references here. If not, I am wondering if it would be worthwhile to check the adaptation by comparing steady-state ISIs. Maybe different neurons have different time course of adaptation but at the end they show same level of adaptation.**
5. **If we run the model for longer duration, how does the behavior change, does it continue to adapt or it reaches a steady-state?**
6. Second paragraph of Strongly adapting model:
*"If these properties (f-I curve, rheobase current and adaptation) are captured, then the model will spike with similar frequencies as the physiological cell given the same amount of synaptic input."* ******
**Is the applied protocol (continuous injections of currents for 1s) sufficient to characterize the response of these neurons in the presence of synaptic inputs?**
7.  Second paragraph of Weakly adapting models:
*"In addition, to obtain an appropriate rheobase current, we included a short in the applied current."* ******
**I do not understand why *I _shift_* term is included in *I _applied_* to adjust rheobase?**
8. Third paragraph of Discussion and conclusion:
*"In previous work, we used adaptation characteristics from the literature to develop simple models of CA3 pyramidal cell models, and showed that population characteristics were in line with the experimental data."*
**It would be interesting and more insightful to see how this work can be extended to network models as has been done previously (in references 6 and 23) by the authors.**
9. Third paragraph of Discussion and conclusion: 
*"Here, with our simple mathematical model representation and knowledge of the biological variability in hand, one is easily aware of any changes in the parameters of the model that would result in large deviations from the experimental data." *
**This is very important aspect. But that would only be possible if the effects of parameters can be characterized. It would be interesting to see any such generalizations.**
** **
10. Fourth paragraph of Discussion and conclusion:
  "Here, we have developed out cellular models in the experimental context for which we build the network models to determine physiologically-based mechanisms." 
**Building network models incorporating CA1 pyramidal cells and determining physiologically-based mechanisms is clearly not presented in this paper and should be indicated as future work.**
11. **In Figure 3, please also show rebound firing of recorded cells, if available.**
12. **In Figure 2,  the label pointing to green dotted line is misspelled. It should be final curve instead of *"finial curve"***.

We have read this submission. We believe that we have an appropriate level of expertise to confirm that it is of an acceptable scientific standard.

# Response

**Published:** 2014-06-24

**Authors:** Frances Skinner

**Affiliations:** University Health Network, Canada

We would like to thank the reviewers for their helpful comments.  We will respond more specifically and revise our article accordingly in the near future.

We would like to point out that we focused on the single cell model and did not try to also include network simulations in this article as it was originally submitted as a 'Short Research Article' type.

However, *F1000Research* recently replaced the 'Short Research Article' category with a shorter 'Research Notes' article type. As this article did not fit the criteria of a 'Research Note', it appears as a 'Research Article' instead.
